# Sutureless Intrascleral Posterior Chamber Intraocular Lens Fixation: Analysis of Clinical Outcomes and Postoperative Complications

**DOI:** 10.1155/2021/8857715

**Published:** 2021-01-16

**Authors:** Jiannan Liu, Wenxue Fan, Xinyu Lu, Shaomin Peng

**Affiliations:** ^1^Aier School of Ophthalmology, Central South University, Changsha, China; ^2^Harbin Aier Eye Hospital, Harbin, China

## Abstract

**Purpose:**

To report a technique for performing sutureless intrascleral fixation of a posterior chamber intraocular lens (PC-IOL) and analyzing the clinical outcomes and postoperative complications. *Study Design*. 68 eyes of 66 patients who received the technique were studied retrospectively.

**Methods:**

The best-corrected visual acuity (BCVA), intraocular pressure (IOP), anterior chamber depth (ACD), IOL tilt and decentration, corneal topography (*K*1 and *K*2), and postoperative complications were determined at 3 months.

**Results:**

The mean preoperative BCVA was 1.63 ± 1.24 logMAR units, and the mean postoperative BCVA was 0.74 ± 0.59 logMAR units at 3 months (*P* < 0.05). The mean preoperative IOP was 21.9 ± 12.6 mmHg, and the mean postoperative IOP was 16.9 ± 4.5 mmHg at 3 months (*P* = 0.001). The mean preoperative corneal topography (K1 and K2) was *K*1 = 42.14 ± 1.91 and *K*2 = 43.54 ± 1.51; the mean postoperative corneal topography (*K*1 and *K*2) was *K*1 = 43.03 ± 2.18 and *K*2 = 43.40 ± 1.71 at 3 months (*P* = 0.678 and 0.468, respectively). The mean preoperative spherical equivalent was +11.00 ± 13.19 diopters (*D*), and the mean postoperative spherical equivalent was +0.06 ± 0.86 *D* (*P* < 0.005). The mean IOL tilt was 2.4 ± 1.7°, and the mean decentration was 0.35 ± 0.21 mm. The mean ACD was 4.31 ± 0.29 mm.

**Conclusions:**

The 27-gauge sutureless intrascleral PC-IOL implantation technique minimizes intraoperative injury, simplifies procedure, and provides good PC-IOL fixation with few postoperative complications.

## 1. Introduction

The absence of sufficient capsule support may happen as a result of incompetence of zonules [1], cataract surgery, vitreoretinal surgery, or following traumatic injury to anterior segment structures. In this situation, the fixation of an intraocular lens (IOL) is challenging and very variable. Various techniques of IOL fixation can be performed including anterior chamber IOL (AC-IOL) implantation, iris fixing posterior chamber IOL (PC-IOL) implantation, and transscleral sutured fixation of a PC-IOL in the ciliary sulcus or pars plana [2, 3].

Transscleral suture fixation through the ciliary sulcus or the pars plana plays a principal role during the operation. But there are some severe postoperative complications in long-term studies [4–13], such as suture exposure, suture degradation, transient hypotony, and endophthalmitis. Gabor and Pavlidi [14] first described a new technique of sutureless intrascleral fixation of a PC-IOL. Agarwal et al. [15] introduced a sutureless intrascleral technique with scleral flaps and fibrin glue. Yamane et al. [16] and Zhang et al. [17] developed the technique of sutureless intrascleral PC-IOL fixation to be more operable and convenient. Takayama et al. [18] advanced the sutureless intrascleral technique without applying a wide conjunctival incision. Barca et al. [19] introduced Carlevale lens to simplify the operation. However, postoperative complications still exist. In this case, we modified the technique and focused on the analysis of clinical outcomes and postoperative complications.

## 2. Methods

68 eyes of 66 patients who had received sutureless intrascleral haptics fixation of the PC-IOL under the 27G vitrectomy system between November 2017 and December 2019 were studied retrospectively. All surgeries were performed using the constellation vitrectomy 27+ vitrectomy system (Alcon Laboratories, Fort Worth, TX) by the same ophthalmologist at the Harbin Aier Eye Hospital, Harbin, China. The surgical protocol was approved by the Institutional Review Committee of Harbin Aier Eye Hospital, and all clinical investigations and procedures were conducted according to the principles of the Declaration of Helsinki of 1975, as revised in 2013. Informed consent was acquired from all the patients after being informed about the process and the possible complications of our technique.

**Figure 1:**
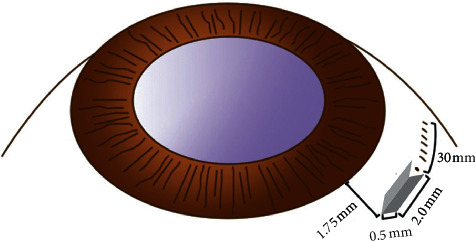
Diagram of position of scleral dissection.

Under peribulbar and subconjunctival anesthesia, a 4 mm × 6 mm fornix-based peritomy was applied, and a standard 3-port 27G pars plana vitrectomy (PPV) was performed. During the operation, dislocated lens have been removed by lensectomy, phacoemulsification, or ultrasonic fragmentation according to the age of patients and hardness of lens nucleus. Then, a 3 mm sclerocorneal incision and two bilateral scleral dissections of 2 mm in length and about 0.5 mm thickness were performed 1.75 mm from the limbus (Figure 1). The two incisions were precisely 180° apart from each other (Video 1). And two scleral punctures were conducted a 27G-stab knife at the bottom of the left incision and top of the right incision with slight extension.

**Figure 2:**
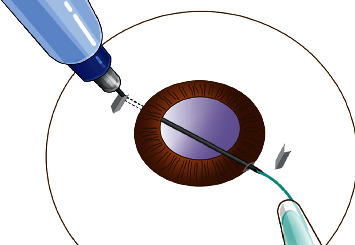
Diagram of the leading haptic guided by the needle.

**Figure 3:**
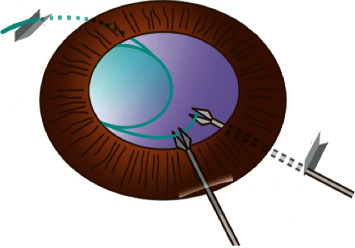
Diagram of the trailing haptic grasped by 27G forceps.

**Figure 4:**
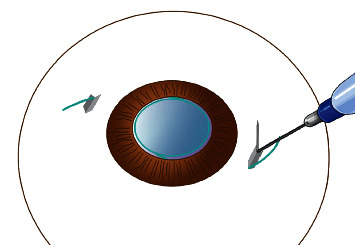
Diagram of the tunnel made by a bent 29G × 1/2″ needle.

**Figure 5:**
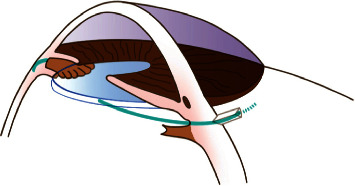
Diagram of position of haptics.

Before, a standard 3-piece folded IOL (AcrySof MA60AC, Alcon or Tecnis ZA9003, AMO) was implanted with an injector through the sclerocorneal incision; a needle of a 1 ml injector was bent at straight angle and was passed from a sclera puncture to sclerocorneal incision. The leading haptic was inserted into the needle and pulled out of the eye (Figure 2), and the trailing haptic was left outside the eye temporarily. The trailing haptic was sent into the anterior chamber by a 27G forceps through the sclerocorneal incision, and the end tip of the trailing haptic was then grasped and pulled out of the eye by another 27G forceps which was put through the right scleral puncture (Figure 3, Video 2). At the top of the left incision and the bottom of the right incision, two 3 mm scleral tunnels parallel to the limbus was prepared counterclockwise by a 29G × 1/2″ needle which has been bent at right angles in advance (Figure 4). Then, both the leading haptic and the trailing haptic were pulled into the scleral tunnel by 27G forceps, respectively (Video 3). The IOL was centered through adjusting the intrascleral position of both haptics (Figure 5). This surgery can be performed combining with perfluoropropan (C_3_F_8_) or silicone oil tamponade for retinal attachment. At last, the infusion cannula can be removed, and conjunctiva can be fixed with 8–0 absorbable sutures.

The best-corrected visual acuity (BCVA), intraocular pressure (IOP), IOL tilt, IOL decentration, anterior chamber depth (ACD), and complications were determined. Scheimpflug imaging (OCULUS PENTACAM) and ultrasound biomicroscopy (UBM) (SW-3200L SUOYA) were performed to evaluate the tilted angle and decentration distance of the IOL and anterior chamber depth (ACD) for each eye and corneal topography (*K*1 and *K*2) 3 months postoperatively. The IOL tilt and decentration were measured and calculated in both the vertical and horizontal planes. The decimal BCVA was converted to the logarithm of the minimum angle of resolution (logMAR) for the statistical analyses. The Wilcoxon signed-rank test was used to determine the significance of any association between the preoperative and postoperative BCVA and IOP. *P* < 0.05 was considered significant. The statistical analyses were performed using the SPSS software (version 22.0 for Windows SPSS, Inc.,Chicago, IL).

## 3. Results

Our surgical study consisted of 68 eyes of 66 patients (47 men and 19 women) with a mean age of 61 years (range 25–82 years), including 27 aphakia with incomplete lens capsule, 8 dislocated PC-IOLs, 22 subluxated crystalline lenses, and 11 luxated crystalline lenses (Tables 1 and 2). 33 lens have been removed with phacoemulsification, 15 lenses with ultrasonic fragmentation, and 13 lenses with lensectomy. The rest were extracted by other surgeons from other hospitals. The mean follow-up was 10 months. There was no postoperative retinal detachment, endophthalmitis, or IOL haptics exposure detected during the follow-up period.

The mean preoperative BCVA was 1.63 ± 1.24 logMAR units, and the mean postoperative BCVA improved significantly to 0.74 ± 0.59 logMAR units at 3 months (*P* < 0.05). The mean preoperative IOP was 21.9 ± 12.6 mmHg, and the mean postoperative IOP was 16.9 ± 4.5 mmHg at 3 months (*P* = 0.001) (Table 3). The mean IOL tilt was 2.4 ± 1.7°, and the mean decentration was 0.35 ± 0.21 mm. The mean ACD was 4.31 ± 0.29 mm. The mean preoperative corneal topography (*K*1 and *K*2) was *K*1 = 42.14 ± 1.91 and *K*2 = 43.54 ± 1.51; the mean postoperative corneal topography (*K*1 and *K*2) was *K*1 = 43.03 ± 2.18 and *K*2 = 43.40 ± 1.71 at 3 months (*P* = 0.678 and 0.468, respectively). The mean preoperative spherical equivalent was +11.00 ± 13.19 diopters (*D*), and the mean postoperative spherical equivalent was +0.06 ± 0.86 *D* (*P* < 0.005). There were transient IOP rise (4.5%), hyphema (3.0%), vitreous hemorrhage (1.5%), macular edema (1.5%), haptic exposure (3.0%), and pupillary capture (8.8%) detected during the follow-up period.

## 4. Discussion

Secondary implantation and refixation of PC-IOL have been performed in patients with deficient capsular support after ocular surgery or trauma. As for AC-IOL implantation, though the postoperative corrected distance visual acuities (CDVA) was not significantly different from PC-IOL implantation [20], endothelial cell loss, pseudophakic bullous keratopathy, glaucoma, and chronic iritis [21–23] are still the main postoperative complications. And for iris-fixated IOL implantation, the main postoperative complications are chronic iritis, endothelial cell loss [24], and pupillary distortion [25]. For transscleral suture fixation, long-term studies revealed that suture exposure rate ranges from 5% to 50% [4–7]. Although, majority of surgeons placed the suture knots subconjunctivally or under a scleral flap [8] to avoid relatively high suture exposure. The ratio remains 14.7%–17.9% [9–11]. Girard [26] described a sutured technique for fixing the IOL through the pars plana; however, the decentration or tilt [27] of PC-IOL depends on suture hypotony or degradation [28], and endophthalmitis due to suture exposure [29] remains to be a problem. Surgeons applied for the Gore-Tex suture method which enables to improve problem of the suture degradation. However, the problem of transient hypotony and suture exposure still exists [13, 30].

Gabor and Pavlidis [14] reported sutureless intrascleral fixation of PC-IOL, in order to avoid the sutured PC-IOL situation and enable the PC-IOL more proximity to the ocular rotational axis and nodal point [31]. In Scharioth's technique, two scleral tunnels about 50% scleral thickness, starting directly from sclerotomies were performed by a 24-gauge cannula, and the haptics were grasped and pulled out through the sclerotomies by a 25-gauge forceps. In our study, we updated the sclerotomies and entrances of scleral tunnels for haptics apart, which enabled the operator to pull the haptics into the tunnels much more easily.

Yamane et al. [16] externalized the haptics of the IOL and inserted the IOL haptics into the scleral tunnel in a more operable and convenient manner, as sclerotomy and scleral tunnel are apart. And two scleral tunnels were conducted by a 27G needle. During our study, the scleral tunnels were prepared by a 29G × 1/2″needle and tightly sealed around the haptics, which stabilized the haptics. The cross-section diameter of a 27G needle is 0.4 mm, while a 29G × 1/2″needle is 0.33 mm and an IOL haptic (Alcon AcrySof MA60AC) is 0.14 mm. The 29G × 1/2″ needle was bent at right angles, and the distance between the tip and the bend point was 3 mm. In our technique, the tunnels are tighter and the stability of IOL is better.

Agarwal et al. [15] introduced that sutureless PC-IOL implantation with glue, in which scleral flaps were performed and developed by Kumar et al. [32–34]. However, a possibility of transmission of viral infections theoretically existed [35], and a large lamellar scleral flap was required. Comparing to Amar and Kumar's technique, ours does less trauma and costs less.

Barca et al. [19] applied a novel one-piece foldable IOL named Carlevale lens which simplified the procedure and shortened the learning curve. And the fixation of Carlevale lens was stable due to the specially designed sclera-corneal plugs. These are all superior to 3-piece IOL sutureless intrascleral fixation technique. Rossi et al. [36] reported a 1.3% incidence of T-shaped Carlevale lens harpoon ruptured when grasped to externalize it in their study of 78 patients. The T-shaped Carlevale lens harpoon was much more diminutive than a 3-piece IOL haptic. In our technique, we applied a needle to guide the leading haptic, and the trailing haptic of 3-piece IOL was easier for surgeons to externalize.

**Figure 6:**
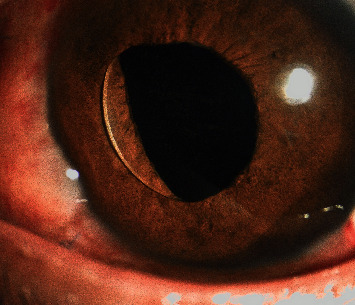
Photography of pupillary capture.

**Figure 7:**
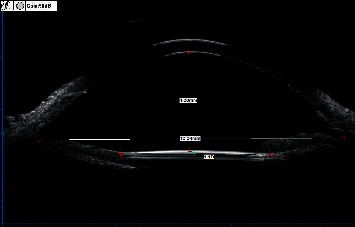
Anterior chamber depth measured by UBM.

Another 6 (8.8%) patients suffered pupillary capture with iris depigmentation (Figures 6 and 7), at 2 weeks after operation. The IOP of three was 16∼18 mmHg and pupil was dilatate. Then, Nd:YAG laser iridotomy was performed on one patient to improve the situation. In 3 studies reported by Kumar et al. [33, 34, 37], the incidence of pupillary capture is 4.30%, 2.63%, and 2.40%, respectively, while Shin et al. [16] and Takayama et al. [18] reported it to be 8.60% and 8.30%. Shin et al. [16] performed a peripheral iridotomy by a vitrectomy cutter to avoid iris capture of the IOL, but pupillary capture remains the highest rate of postoperative complication in their study. There was no pupillary capture occurred in studies of Carlevale lens [19, 36, 38, 39]. There is a 5° anterior angulation relative to the optic plate of Carlevale lens haptics to ensure enough space between iris and IOL, which may minimize iris chafing and pupillary capture. During the surgery, the two conditions might happen: pupillary dilatation on account of the injured sphincter pupillae muscle [40, 41] and IOL decentration owing to asymmetric fixation [42]. After the surgery, the removal of the lens capsule and the lens zonular fibers with vitreous preserved a greater posterior chamber volume resulted in iris backward and more aqueous humor flowing into the anterior chamber [43, 44] to push IOL up. And iris depigmentation due to the contact between IOL and the iris pigment epithelium might cause angle pigmentation, which slowed the aqueous humor flowing out from the anterior chamber [45]. We speculated these four reasons are responsible for pupillary capture. In our study, the mean ACD was 4.31 ± 0.29 mm, which comparing to 4.47 ± 0.31 mm in capsular bag reported by Hoffer and Savini [46] was a little shallower. Choi et al. [42] reported the ACD was significantly shallower in the eyes with IOL capture, while Kang and Kim [47] reported that a larger ACD increased the risk for pupillary capture. Therefore, further study of large sample is essential to explain whether there is the potential relationship between pupillary capture and ACD.

In the study, four eyes were diagnosed as intraocular hypertension preoperatively, including three eyes that suffered vitreous prolapse and one eye that suffered traumatic hyphema. Vitreous and blood might occlude the angle of the anterior chamber and increase intraocular pressure for the patients. The 27G vitrectomy system represented that its cutter is sufficient to cutoff vitreous and blood in the angle.

Two patients (2.9%) suffered hyphema and one patient (1.5%) suffered vitreous hemorrhage on the first day after surgery. There were some small patches of blood on the iris and a little bit of blood in the vitreous chamber. After 2 or 3 days, blood was absorbed, and neither hyphema nor vitreous hemorrhage appeared during the next follow-up period.

The haptics of two high myopia patients (2.9%) came out postoperatively, as the sclera of theirs was thinner than normal people. We had to suture the scleral dissections and tunnels. So, we suggest that when dealing with patients with high myopia, surgeons should be careful of the thin sclera. And it is reported that maybe it is not appropriate for the eyes with thin sclera, scleritis, areas of scleromalacia, and staphyloma [48, 49].

One special case presented that dissections and tunnels were performed superior and inferior and precisely 180° apart from each other. This was different from horizontal dissections of other cases and IOL stability remains.

A 25-year-old female Marfan's syndrome patient who suffered from dislocated crystalline lens received the fixation. It is more beneficial to young patients to apply for our technique, as less trauma and self-sealing sclerotomy injury.

Taken together, the 27-gauge sutureless intrascleral PC-IOL implantation technique with 29Gi1/2″ needle minimizes intraoperative trauma, simplifies procedure, and provides good PC-IOL fixation with few postoperative complications currently. Although sutureless intrascleral PC-IOL fixation were reported previously, the long-term anatomical and functional stay would be considered.

## Figures and Tables

**Table 1:** Indications for surgery.

	Trauma	Incompetence of zonules	Cataract surgery	Vitreoretinal surgery
Aphakia with incomplete lens capsule	13	2	6	6
Subluxated crystalline lenses	12	7	2	1
Luxated crystalline lenses	4	7	0	0
Dislocated PC-IOLs	3	5	0	0

**Table 2:** Characteristics.

	Ocular hypertension	Vitreous prolapse	High myopia	Traumatic hyphema	Silicone oil filled eye
Aphakia with incomplete lens capsule	5	0	0	0	9
Subluxated crystalline lenses	6	4	1	2	0
Luxated crystalline lenses	3	2	2	0	0
Dislocated PC-IOLs	0	0	3	0	0

**Table 3:** Comparison of BCVA and IOP before and after management.

	Preoperative	Postoperative (3 months)
BCVA (logMAR units)	1.63 ± 1.24	0.74 ± 0.59
IOP (mmHg)	21.9 ± 12.6	16.9 ± 4.5

## Data Availability

The datasets analyzed during the current study are available from the corresponding author upon request.

## Abbreviations

BCVA: Best-corrected visual acuity
IOL: Intraocular lens.
PC-IOL: Posterior chamber intraocular lens
AC-IOL: Anterior chamber intraocular lens
IOP: Intraocular pressure
ACD: Anterior chamber depth
D: Diopters
PPV: Pars plana vitrectomy
UBM: Ultrasound biomicroscopy
CDVA: Corrected distance visual acuities.

## Supplementary Materials

Supplementary Materials Video 1. Determining the position of IOL (). Video 2. IOL implantation (). Video 3. Haptics of IOL fixation ().
