# Supplementary material for: Sutureless Intrascleral Posterior Chamber Intraocular Lens Fixation: Analysis of Clinical Outcomes and Postoperative Complications
Source: J Ophthalmol. 2021 Jan 16;2021:8857715. doi: 10.1155/2021/8857715 (PMC7826223; doi:10.1155/2021/8857715)
Supplement: Supplementary Materials — Video 1. Determining the position of IOL (). Video 2. IOL implantation (). Video 3. Haptics of IOL fixation (). [file 8857715.f1.docx]

Supplementary file can be downloaded from this link:

[https://drive.google.com/drive/folders/1Pui4jqQNzewhl-Nu-8IRZ4WJZmS9Mmq4?usp=sharing](https://www.google.com/url?q=https://drive.google.com/drive/folders/1Pui4jqQNzewhl-Nu-8IRZ4WJZmS9Mmq4?usp%3Dsharing&sa=D&source=hangouts&ust=1609413451824000&usg=AFQjCNH_2l4W41OdIBPMWJ-jmu9ztyPe4A)
